# Surgical treatment of spasticity: intrathecal baclofen pump implantation under subarachnoid block

**DOI:** 10.3171/2020.7.FOCVID2021

**Published:** 2020-10-01

**Authors:** Alba Scerrati, Pasquale De Bonis, Nicolò Norri, Michele Alessandro Cavallo

**Affiliations:** Department of Neurosurgery, Ferrara University Hospital, Ferrara, Italy

**Keywords:** spasticity, intrathecal baclofen pump, subarachnoid block, video

## Abstract

Most patients with spasticity, rigidity, and other symptoms of the upper motor neuron syndrome respond effectively to surgical treatment with an intrathecal baclofen pump when their symptoms become intractable to nonsurgical measures. Baclofen administered into the lumbar subarachnoid space produces a locally high concentration at the spinal level and a low concentration supraspinally, avoiding most of the central side effects associated with a high oral dose, such as drowsiness and confusion.

The aim of surgical treatment is to provide the appropriate volume and concentration of the drug in the subarachnoid space, avoiding the main surgical complications, that is, infections, skin erosion, and catheter displacement.

The video can be found here: https://youtu.be/HetelPwwwak

## Transcript

This video describes the surgical procedure of intrathecal baclofen pump implantation for the treatment of spastic paresis.

**0:27** This patient is a 43-year-old woman who was diagnosed with multiple sclerosis at the age of 22 years old.^1^ In January, the patient was admitted to the hospital with clinical signs of spastic tetraparesis with a greater involvement of lower limbs.

Patient was tested with 50-µg intrathecal baclofen infusion by lumbar puncture and showed improvement of her Ashworth score and Penn score.^2,3^ Thus, she underwent surgery for a baclofen programmable pump implantation.

**0:55** One hour before the operation starts, we administer 1 g of intravenous vancomycin as antibiotic prophylaxis.

**1:02** In the surgical theater, the patient is positioned on lateral decubitus and spinal anesthesia is performed. The patient is positioned supine while waiting the anesthetic to be effective.

**1:12** We furtherly position again the patient in a right lateral decubitus.

**1:19** We carefully disinfect the skin with chlorhexidine gluconate and prepare the surgical field, paying attention to leave uncovered the back, the left side, and the homolateral paraumbilical region.

**1:30** Before the skin incision, we identify the right interlaminar L3–4 and L4–5 space by touching the spinous processes of L3–L4–L5. We mark them and we perform local anesthesia before skin incision over the midline. We open the cutaneous and subcutaneous layers in order to expose the lumbar fascia. Although many surgeons have advocated for a paramedian approach, we usually obtain access through the midline.^4^

**1:53** We position the 15-gauge Tuohy needle through the L4–5 interlaminar space, making sure that the CSF comes out, and we push forward the spinal catheter in the subdural space for about 20 cm. It is essential to fill the entire system of catheters with physiological solution in order to remove the air, thereby preventing their obstruction.

**2:18** After removing the stylet of the catheter and checking again the outflow of CSF, we anchor the spinal drainage to the muscular fascia using the specific fixation wing in order to avoid catheter dislocation and at the same time paying attention not to close the system.

**2:49** The preparation of a new pump includes three main phases. The first one consists of inserting the needle of an empty 50-cc syringe into the reservoir fill port in order to extract a predetermined volume of the fluid contained in the device; it is essential not to remove the needle before the air bubbles start coming out in the syringe.

**3:08** The second one consists of filling the empty pump with a 50-cc syringe containing 40 cc of baclofen drug.^5^

**3:16** The last key step, which can only be done with the programmable pumps, is the calibration and adjustment of the releasing dose, rate, and timing parameters of the pump, using an external programmer.

**3:28** The surgeon incises the cutaneous and subcutaneous layers of the left paraumbilical region until the superficial fascia is exposed.

**3:39** A new small skin incision is made on the side, in order to allow the passage of the extension catheter with the trocar. The extension catheter is double-tunneled under the skin. The first step consists of pushing the trocar from the side incision toward the abdominal pocket; the second one, of passing the catheter from the side toward the back.

**3:58** Now the catheter connector, part of the extension system, can be inserted inside the spinal catheter and tied with a knot of unabsorbable suture.

**4:22** After the connection of the extension catheter to the catheter port of the pump, the whole system is ready, and the pump is placed inside the abdominal pocket. On the sides of the pump, several suture loops are used to secure it to the abdominal fascia. We always try to leave an appropriate subcutaneous layer in closing the skin and to release possible tensions, in order to prevent skin erosion and decubitus.^6^

**4:45** It’s essential to close very carefully the subcutaneous and cutaneous layer, in order to prevent infections and dehiscence of the wounds. Usually, we remove stitches 2 weeks after surgery.

## Author Contributions

Primary surgeon: Cavallo. Assistant surgeon: Norri. Editing and drafting the video and abstract: Norri. Critically revising the work: Scerrati, De Bonis, Cavallo. Reviewed submitted version of the work: all authors. Approved the final version of the work on behalf of all authors: Norri. Supervision: Scerrati, De Bonis, Cavallo.
